# Dr. Harrison's Case of Hydrophobia

**Published:** 1807-09

**Authors:** J. Harrison

**Affiliations:** Milford, S. W.


### *To the Editors of the Medical and Physical Journal.*

GENTLEMEN,

READING in your Journal for May last, the Address to the Professors of Physic and Surgery, for investigating the Cause, Symptoms, &c. for the Cure of Hydrophobia, and wishing to be useful to mankind, I beg leave to relate a case in the fewest and plainest words possible. A dog, not supposed to be mad, came into the yard of Mr. J. Stoll, farrier, at Charlestown, South Carolina; he bit several hogs, a cat, a woman, and a boy, about fifteen or sixteen.

sixteen. The hogs were first seized with the rabies and died raving mad, as well as the cat. The boy was next attacked with the hydrophobia; I was directly sent for, and found him in a very dreadful situation, endeavouring to bite every thing. Upon the sight of a glass of tea, he was seized with the most violent convulsions I ever beheld. He was copiously bled, and the intestinal canal emptied with cathartic injections. As I had not seen many cases of rabies, I requested a consultation with Dr. John Delapowe, one of the most learned, skilful, and ingenious physicians I ever had the honor of knowing. He advised the part to be scarified and cauterized with a red hot iron, and dressed with blistering ointment; which was immediately done. We retired into a private room, where Dr. Delapowe made the following observations. The hydrophobia is a spasm, not only of the pharynx but of the œsophagus also, and the dread of choking, when shown any liquid, is mental. Therefore I propose that we should endeavour to change his ideas, by taking him in a boat, out of his depth in the sea, and detaining his head under water, until he is *all but dead*. I undertook to superintend this business myself, and kept him under water until he was totally deprived of his senses, and indeed I feared, *not a little*, that he could not be restored to life. However, with frictions, &c. in an hour, or thereabouts, he opened his eyes, and sometime afterwards spoke. He of course must have swallowed some sea water; but his ideas of the dread of liquids were removed. He drank a little grog; rested tolerable that night. The next day he ate and drank moderately, and slept very easy the following night. The third day he appeared to be perfectly well, and his sleep was natural, without any symptoms of the disease; we now congratulated ourselves that we had discovered a method of cure after the commencement of the dread of water. Alas! the fourth day, about noon, he was seized with a violent vomiting, which resisted the power of medicine. He expired quite exhausted, on the seventh day, without the least symptom of the hydrophobia.

Q. What did this vomiting proceed from? The bowels were open: He took broth, gruel, &c. &c. but could not retain any thing on his stomach.

The woman went through a mercurial course and escaped. She was kept in a gentle ptyalism three weeks. This happened in the year 1774.

I am, &c.

*Milford, S. W. July* 4, 1807. J. HARRISON. M. D.

P. S. I have vaccinated (gratis) more than 150, in defiance of one of the most illiberal, malicious combinations, of country practitioners, that any Gentleman ever met with. I have also to observe, that several of the vaccinated children lay in the same beds with other children infected with the small-pox, without taking the disease.

